# Clinical Reports of Diseases, Injuries, Surgical Operations, Etc., Etc., at the Buffalo Hospital of the Sisters of Charity

**Published:** 1857-09

**Authors:** Benj. H. Lemon

**Affiliations:** Formerly House Surgeon


ART. V.— *Clinical Reports of Diseases, Injuries, Surgical Operations, &c., &c., in the Male and Female General Surgical and Private Wards of the Buffalo Hospital of the Sisters of Charity.* Care of Prof. HAMILTON, for the winter half year commencing Oct. 1st, 1856. By BENJ. H. LEMON, M. D., formerly House Surgeon.

(Continued from Page 167.)

CASE VII.—*Ptosis. Operation, and good result.*

Dr. ——, æt. 23 years, lately graduated in Toronto. Has falling of the eyelid; been in this state many years; came on after a splinter had run through the palpebra. It covers about two-thirds of the globe, when opened to the utmost extent of his ability. The tarsus is everted, and cellular tissue thickened in consequence of an unsuccessful operation.

*Diagnosis.* Falling of the lid in consequence of paralysis of the levator palpebræ, caused by injury to its motor nerve, "the distribution of the superior branch of third pair."

Operation was made by excising a portion of integument from the superior part of the vertical diameter of the lid, about six lines in width, thus bringing the palpebra somewhat under the influence of the occipito-frontalis, by taking out the skin at the upper margin of the lid, a tendency to greater eversion of the tarsus was avoided. Three sutures were applied and a pledget of lint wet with cool water.

The tarsus will now come about one line below the superior margin of the cornea, when open; and when shut a narrow line can be seen at the center, not sufficient to cause inconvenience.

At his own urgent request chloroform was given, which made him very unruly, he having to be held.

In the operation for ptosis, it is desirable to remove enough to secure a sufficient elevation of the eyelid without at the same time taking as much as will prevent closure and coaptation of the tarsi, because if the fissure between them was wide, the globe of the eye would become dry and injured. As a general rule the amount required to be removed is greater than would be generally supposed; tлis is one of the two errors into which the first operator fell, namely, first, not removing the integument extensively enough; and secondly, not removing that portion in the proper place, but taking it out at the tarsal side of the median transverse line, thus causing eversion.

Case VIII.—*Subacute Synovitis, with effusion; left Knee-joint. Recovery, with impaired motion.*

There was a number of cases of this character; all, except the two cases I shall report, recovered entirely the use of their limbs.

John McGarty, æt. 30 years; laborer; of nervous temperament, dark complexion. Admitted Dec. 8th. He works in a grain elevator; is exposed to cold. His work in shoveling causes him to twist around; the condyles of the femurs rotate on the articular surfaces of the tibias, while the feet are embedded in the grain and do not move, thus straining the ligaments and subjecting the articular surfaces to actions they were not intended to subserve, the joint being purely ginglymoid, and any other motions unnatural, and injurious.

The patient took cold, and six days ago while at work, he felt sharp pain in the left knee, which increased so much that he was obliged to go home. The limb became swelled, and the joint stiff: motion causing pain. The region of the joint is much enlarged, chiefly at the sides, and above the patella, caused by effusion in the sac of the synovial membrane.

*Diagnosis.* Subacute synovitis, with hydrarthros.

*Treatment.* The dorsal-recumbent posture; the limb extended, and the joint supported by a pillow; antiphlogistic regimen; tincture of iodine painted on and around the region of the joint, and occasionally a blister to be applied.

He soon required supporting by ale, quinia, &c. He slowly improved, however, suffering much from the pain in the joint, particularly at night.

He continued in the house until the close of Prof. Hamilton's service, the joint gradually improving.

I saw him after he had been discharged, sometime in May; he could not yet bear any weight upon the limb; it was considerably larger than the sound one, about the joint; the muscles, both above and below, were much emaciated; motion was much impaired; doubtless, however, the size will become much reduced, and a considerable amount of action restored with the lapse of time, as his general health and strength improves, and ultimately a good and useful limb may be expected.

"Sir Benj. Brodie" records ten cases of synovitis in the knee-joint. In five, there was recovery, with impaired motion of the joint. In one, no stiffness remained; and in four, he does not state whether there was this defect or not.

As to causes, scrophula seems the predisposing, and exposure to cold, dampness, sprains, injuries, &c, the exciting. It commences by the usual phenomena of inflammation, a preternatural secretion of synovial fluid, effusion of coagulable lymph in the cavity of the joint, if not checked suppuration of the synovial membrane, collections of pus in the cavity, which, with serum and lymph is effused into the tissues external to the joint, the pus burrowing under the fasciæ and tendons, forming cavities and sinuses; finally, softening, degeneration, and ulceration of the coagulated lymptb, cartilages and ligaments, and total destruction of the joint results, which the following case will illustrate.

Case IX.— *Synovitis, Ulceration of the Synovial Membrane, Cartilage and Ligaments, and Caries of the Articular Surfaces, and Cancellous Tissues of the Bones of the Knee-joint. Amputation at the middle of the Thigh. Morbid appearances. Death of the patient in six weeks.*

Thomas Walsh, æt. 28 years, stout, laborer. Admitted Jan. 28th. The winter of 1854, whilst walking on the railway, he fell, striking the inside of his left knee on the iron rail, hurting it severely; swelling very soon occurred, and the joint became stiff and sore, and he was unable to bear weight on the limb all winter; by the summer it was sufficiently well for him to go about, and he began hard work in the freight house. The winter following it again inflamed, this time without any external cause; it soon recovered.

The summer of '55 this joint was again hurt on the inside, and he was a cripple for several weeks; the inflammation finally disappeared, but left the knee larger than that of the sound limb, and he returned to his duty on the railway.

Oct., 1855, he received a heavy blow from a plank, on the outside of his knee. The joint grew inflamed and swelled to a larger size than it had at any previous time. Motion was entirely lost, and the pain extreme. The symptoms gradually became worse, and he has been incapacitated for any work since.

*Present State.* Lies on his back, the limb extended, and knee supported with a pillow. The joint is much, and unequally enlarged; the leg œdematous, pitting under pressure of the fingers; muscles of the thigh emaciated, (as indeed is the whole body); partial luxation of the tibia backward on the femur, (the consequence of softening and relaxtion of the ligaments); crepitus on manipulation, an abscess pointing to the external side, and below the patella; general constitutional disturbance: namely, hectic fever, loss of appetite, night sweats, want of sleep, great pain, emaciation, and general irritation.

The abscess being opened, a large quantity of unhealthy, thin, bloody pus, with cheesy flakes, was poured out, greatly relieving the patient for a time: sustaining treatment with poultices to the knee. Being informed that he would have to lose his limb, he said death was preferable.

12th. Abscess having pointed on the inside, was lanced; from this time the flow of pus was increased, of the same character described above, and in quantity about one pint in twelve hours. He cannot sleep without large doses of opium, and all the symptoms are aggravated, though he has been all the time on full sustaining treatment.

21st. Is much weaker, and in view of speedy dissolution, is desirous of availing himself of any chance an operation may afford, of preserving life.

In consultation with Dr. White, and several other gentlemen, the operation was decided upon, although the result was considered very doubtful.

The same day, (Feb. 22d,) in presence of a number of professional gentlemen, and the class from the Medical College, assisted by Dr. Boardman and others, Dr. Hamilton amputated at the middle of the thigh, without chloroform, the patient preferring the operation without anæsthesia; effusion of blood was very slight; he seemed, however, considerably prostrated after being placed in bed, but rallied with the exhibition of brandy and water.

Dr. Boardman took charge of the patient. Joint examined immediately.

*Pathological Observations.* On laying open integuments found numerous large sinuses filled with pus. Cavity of the joint being laid open, it was also filled with pus. Of the synovial membrane, some portions were thickened, others entirely gone. The crucial ligaments had entirely disappeared, and also most of the semi-lunar cartilages. The lateral ligaments were softened and easily detached from the bones; articular surfaces of condyles of the femur, and the tibia were carious, and the cancellous tissue excavated, resembling honeycomb. The ends of the bones were preserved, and are now in Prof. Hamilton's cabinet.

"Brodie" records five cases in which death resulted from this disease, and five more in which the limb was amputated, in all which the pathological observations are recorded.

Of the history of the patient subsequent to the operation, I am ignorant, save that the wound began slowly to heal, by granulation and secondary adhesion. Subsequently collections of pus in various localities, and death six weeks after the operation.

